# Aspirin induces cell death by directly modulating mitochondrial voltage-dependent anion channel (VDAC)

**DOI:** 10.1038/srep45184

**Published:** 2017-03-22

**Authors:** Debanjan Tewari, Dhriti Majumdar, Sirisha Vallabhaneni, Amal Kanti Bera

**Affiliations:** 1 Department of Biotechnology, Bhupat and Jyoti Mehta School of Biosciences Building, Indian Institute of Technology Madras, Chennai 600036, India

## Abstract

Aspirin induces apoptotic cell death in various cancer cell lines. Here we showed that silencing of VDAC1 protected HeLa cells from aspirin-induced cell death. Compared to the wild type cells, VDAC1 knocked down cells showed lesser change of mitochondrial membrane potential (Δψ_m_), upon aspirin treatment. Aspirin augmented ATP and ionomycin-induced mitochondrial Ca^2+^ uptake which was abolished in VDAC1 knocked down cells. Aspirin dissociated bound hexokinase II (HK-II) from mitochondria. Further, aspirin promoted the closure of recombinant human VDAC1, reconstituted in planar lipid bilayer. Taken together, these results imply that VDAC1 serves as a novel target for aspirin. Modulation of VDAC1 is possibly associated with the cell death and anticancer effects of aspirin.

Aspirin, a nonsteroidal anti-inflammatory drug (NSAID) is widely used as an antipyretic and analgesic agent^1^,^2^,^3^. Aspirin is highly recommended for preventing stroke and ischemic heart attack^4^,^5^,^6^. Although many effects of aspirin are linked with its ability to inhibit cyclooxygenase (COX), a key enzyme in prostaglandin biosynthesis, COX-independent effects have also been reported^2^,^7^. Aspirin has a protective effect against different types of cancer^8^,^9^,^10^,^11^. It induces cell death in different cancer cell lines, such as colon cancer cells, chronic lymphocytic leukemia cells, myeloid leukemia and HeLa cells^12^,^13^,^14^,^15^. Depending on the cell types, aspirin may stimulate apoptosis by activating caspases, up-regulating several pro-apoptotic proteins like Bax, down-regulating Bcl-XL, or targeting NF-κB pathway^15^,^16^,^17^,^18^.

Voltage-dependent anion channel (VDAC) of mitochondria participates in the exchange of ions and metabolites between cytoplasm and mitochondria^19^,^20^. At lower membrane potentials (~−20 mV to +20 mV) VDAC remains open, but it adapts closed conformations with increasing voltages^21^. Unlike other channels, VDAC rarely exhibits fully non-conducting ‘closed state’. Therefore, ‘closed state’ for VDAC often refers to a minimum conductance state which is still permeable to small molecules. In the open state VDAC shows anion selectivity which shifts to cation in the closed state^21^,^22^. VDAC plays a crucial role in the cell survival and death^23^. Opening of mitochondrial permeability transition pore (MPTP), which is associated with the mitochondria mediated apoptosis is believed to be initiated by the Ca^2+^ entry through VDAC1^19^,^24^,^25^,^26^. Although several earlier reports suggested that VDAC is a component of MPTP, recent reports argued against it^27^,^28^. VDAC1 interacts with IP3 receptor (type 3) of endoplasmic reticulum (ER) to transfer low amplitude apoptotic Ca^2+^ to mitochondria^29^. VDAC also interacts with many pro-apoptotic and anti-apoptotic proteins, metabolic enzymes such as hexokinase I/hexokinase II (HK-I/HK- II) and cytoskeletal proteins^30^,^31^,^32^,^33^,^34^. These proteins have been reported to modulate the channel activity. In many cancers e.g. breast cancer, colon lymphoma, prostate cancer and gastric adenomas, HK is over-expressed^35^,^36^. In cancer cells, large fraction of HK is translocated to the mitochondria and interacts with VDAC. Association of HK with VDAC drives the cancer cells towards anaerobic metabolism for compensating higher energy demand^37^. Several compounds e.g. 3-bromopyruvate and methyl jasmonate which are known to dissociate HK from mitochondria have anti-cancer activities^38^,^39^. VDAC1 based peptide induces apoptosis by releasing bound HK from mitochondria^40^,^41^. VDAC is also a target for several pro-apoptotic compounds like curcumin, arsenic trioxide and cannabinoid^42^,^43^,^44^.

In the present study we have identified VDAC1 as a target for aspirin. Aspirin induces closing of purified VDAC1, reconstituted in planar lipid bilayer (PLB). In HeLa cells, aspirin alters cellular Ca^2+^ homeostasis, dissipates mitochondrial membrane potential (Δψ_m_), dissociates HK-II from mitochondria and promotes cell death. Possibly, these effects are manifested by the direct aspirin-induced inhibition of VDAC1.

## Results

### VDAC1 is associated with aspirin-induced cell death

**Figure 1:**
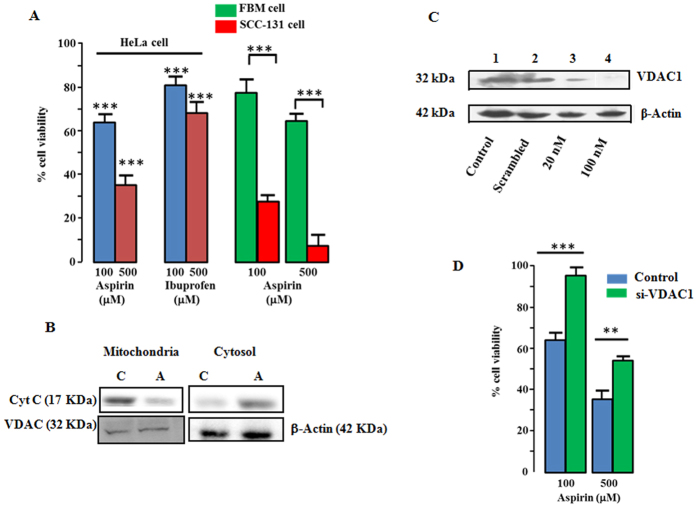
Knocking down of VDAC1 decreases the aspirin-induced cell death . (**A**) Aspirin and Ibuprofen at a concentration of 100 μM and 500 μM decreased the viability of HeLa cells significantly. However, cell death caused by ibuprofen is significantly lesser than aspirin. Effects of aspirin on non-cancerous oral mucosal cell line FBM, and oral cancer cell line SCC-131 are compared. Cell viability reduced more at both 100 and 500 μM concentrations of aspirin in SCC-131 cells. (**B**) Western blot, showing the release of cytochrome c (Cyt C) from mitochondria, upon aspirin treatment. Aspirin-treated cells exhibited lesser amount cytochrome c in mitochondria and higher in cytosol; A: aspirin-treated, C: control. Lower panel shows the loading control, VDAC for mitochondria and β-actin for cytosol. (**C**) Western blot showing the expression level of VDAC1 after siRNA transfection. 100 nM of siRNA suppressed the VDAC1 expression significantly (lane 4). Scrambled siRNA (100 nM; lane 2) had no effect. Lower panel: β-actin band is shown, as loading control. After developing the blot for VDAC1, it was stripped and probed with anti-β-actin antibody. **D.** Effects of aspirin on scrambled siRNA (si-Scr) treated control and VDAC1 knocked down (si-VDAC1) cells are compared. si-VDAC1 transfected cells showed significantly higher viability. Values are the mean ± SEM of five independent experiments.

Aspirin is known to induce apoptotic cell death in different cancer cell lines. When HeLa cells were treated with 100 μM and 500 μM of aspirin for 16 h, cell viability decreased to 64 ± 5% and 35 ± 7% respectively (Fig. 1A). HeLa cells were also treated with another NSAID, ibuprofen. Interestingly, in similar experimental conditions, cell death caused by ibuprofen was substantially lower as compared to aspirin. Cell viability was about 80% and 70% when treated with 100 μM and 500 μM of ibuprofen respectively (Fig. 1A). To test whether aspirin preferentially target cancer cells, its effect on oral cancer cell line, SCC-131 and non-cancerous oral mucosal cell line, FBM were compared. As shown in Fig. 1A, 100 μM and 500 μM of aspirin caused significantly lesser death of FBM cells. Further, we checked the release of cytochrome c from mitochondria in HeLa cells to establish the induction of apoptosis by aspirin. Cells were treated with 100 μM of aspirin for 6 h. Cytosolic and mitochondrial fractions were separated and probed for the presence of cytochrome c by Western blot. In agreement with previous reports^45^,^46^, aspirin treatment decreased the content of cytochrome c in mitochondria and subsequently it was increased in cytosol, confirming the induction of apoptosis (Fig. 1B). The involvement of VDAC1 in aspirin-induced cell death was studied by suppressing its expression with siRNA. To determine optimum dosage of siRNA, cells were transfected with varying concentrations of siRNA and the VDAC1 level was checked after 72 h, using Western blot. 100 nM of siRNA attenuated the VDAC1 expression significantly, while scrambled siRNA had no effect (Fig. 1C). Effect of aspirin was studied on siRNA transfected [si-VDAC1] and scrambled siRNA (si-Scr) transfected cells. Cells were treated with 100 and 500 μM aspirin for 16 h. As shown in Fig. 1D, si-VDAC1-treated cells showed significantly higher viability after aspirin treatment, compared to the si-Scr-treated control cells, suggesting a possible role of VDAC1 in aspirin mediated cell death.

### Aspirin alters mitochondrial Membrane potential (Δψ_m_)

**Figure 2:**
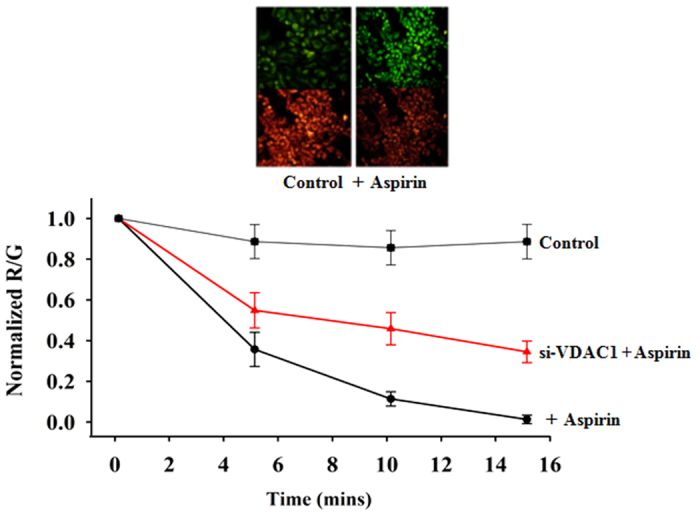
Aspirin decreases mitochondrial membrane potential (Δψ_m_). JC-1 loaded cells were illuminated with 488 nm excitation and the emission was captured at 525 nm (green) and 590 nm (red). Images were acquired at every 5^th^ minute and continued till 15^th^ minute. Decrease in red fluorescence intensity reflects dissipation of Δψ_m_. The change in fluorescence intensity ratio (R/G) is plotted against time. Δψ_m_ of the control cells were maintained while the aspirin-treated cells showed loss of Δψ_m_, as indicated by the fall of R/G ratio. VDAC1 knocked down cells (si-VDA1) showed lesser loss of Δψ_m_ compared to the control. Images, captured at 15^th^ minute are presented.

Apoptosis is often accompanied by a decrease of Δψ_m_. We studied the effect of aspirin on Δψ_m_ in si-Scr-treated and si-VDAC1-treated cells, using JC-1 dye. In healthy mitochondria, the dye aggregates and shows an emission maxima of 590 nM (red). The loss of Δψ_m_ leads to the monomerisation of the dye and emission shifts to 525 nM (green). Thus the decrease in the ratio of red/green fluorescence reflects loss of Δψ_m_. JC-1 loaded cells were treated with 100 μM aspirin and the images (red and green fluorescence) were captured at 5 min interval for 15 min. The Δψ_m_ of control cells (without aspirin) remained unaltered throughout the experimental period. Aspirin decreased the Δψ_m_ in both si-Scr-treated and si-VDAC1-treated cells (Fig. 2). Interestingly, the extent of reduction is much lesser in si-VDAC1-treated cells, compared to the si-Scr-treated cells.

### Aspirin disrupts cellular calcium homeostasis

**Figure 3:**
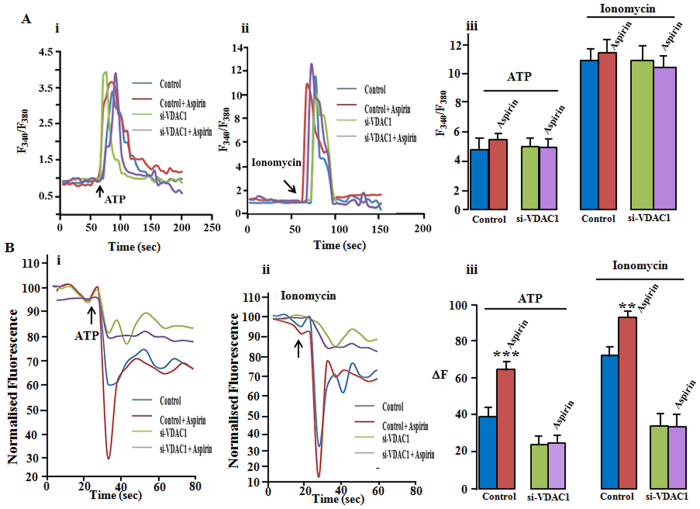
Aspirin enhances mitochondrial Ca^2+^ uptake by targeting VDAC1. (**A,**i) Aspirin did not alter ATP-induced cytosolic Ca^2+^ rise. Both si-Scr treated control and VDAC1 knocked down cells (si-VDAC1 treated) showed similar rise of calcium in response to ATP or ATP + aspirin. Cells were pretreated with Aspirin (100 μM) for 10 min and Aspirin was also co-applied with ATP. In control group, cells were not exposed to aspirin and Ca^2+^ was elevated with ATP alone. ii Experimental conditions are same as i, except ionomycin was applied instead of ATP. Ionomycin caused a bigger increase of [Ca^2+^]_i_. Aspirin had no effect on ionomycin- induced Ca^2+^ rise, either on control or si-VDAC1 treated cells. (iii) Data summary of the results obtained from randomly chosen 25 -30 cells from 3 independent experiments. Values are the mean ± SEM. (**B**) Effect of aspirin on mitochondrial Ca^2+^ uptake. Cells were transfected with mitochondrially targeted inverse pericam. Fluorescence intensity of inverse pericam decreases with increasing Ca^2+^. (i) In control cells, aspirin augmented ATP-induced Ca^2+^ rise significantly. Knocking down of VDAC decreased the Ca^2+^ rise and potentiating effect of aspirin is lost. ATP with or without aspirin elevated mitochondrial Ca^2+^ to the same extent in si-VDAC1 treated cells. (ii) Aspirin potentiated ionomycin-induced Ca^2+^ rise in control cells but not in si-VDAC1 treated cells. Control cells were transfected with scrambled siRNA. The point of ATP/ionomycin application is shown with arrow. (iii) Data summary of 25–30 cells from 3 independent experiments. Experimental conditions were similar to A. **p < 0.01, ***p < 0.001.

Apoptosis is often preceded by the disruption of cellular calcium homeostasis. Aspirin has been shown to increase cytosolic calcium ([Ca^2+^]_i_) in different cell types^47^,^48^. We measured ([Ca^2+^]_i_ ratiometrically using fura-2. As shown in Supplementary Fig. S1, 100 and 500 of μM aspirin did not alter the [Ca^2+^]_i_. We studied the effect of aspirin on ATP and ionomycin-induced Ca^2+^rise. ATP increases Ca^2+^ influx by stimulating ionotropic purinergic receptors. It also releases stored Ca^2+^ from ER by activating *metabotropic purinergic receptors - IP3- IP3 receptors* cascade. Thus, ATP-induced [Ca^2+^]_i_ rise is a combination of Ca^2+^, entered from extracellular solution and released- Ca^2+^ from internal store. HeLa cells have been reported to express both ionotropic and metabotropic purinergic receptors^49^. 1 mM ATP increased [Ca^2+^]_i_ in HeLa cells, as reflected by ~ 4 fold rise of F_340_/F_380_ (Fig. 3Ai,iii.). Cells treated with 100 μM aspirin + ATP showed [Ca^2+^]_i_ rise to the same extent as ATP alone. Further, we checked if VDAC1 is involved in this process. As shown in Fig. 3Ai and iii, both in si-Scr-treated control cells and si-VDAC1-treated cells, [Ca^2+^]_i_ increased to the same extent when treated either with ATP or ATP + aspirin. Calcium ionophore, ionomycin caused a robust rise (~ 10 fold) of [Ca^2+^]_i_. si-Scr-treated control and si-VDAC1-treated cells showed the rise to the same extent (Fig. 3Aii and iii) and aspirin (100 μM) did not alter it.

We measured mitochondrial calcium ([Ca^2+^]_m_) using mitochondrially targeted inverse pericam. The fluorescence intensity (ΔF) of inverse pericam decreases with increasing concentration of [Ca^2+^]_m_. Unlike [Ca^2+^]_i_, cells treated with 100 μM aspirin showed significantly higher [Ca^2+^]_m_ rise in response to ATP (Fig. 3Bi,iii). The ΔF of inverse pericam decreased ~ 40% when 1 mM ATP was applied, whereas the decrease was ~70% in case of aspirin treated cells. In si-VDAC1-treated cells mitochondrial Ca^2+^ uptake is impaired, as anticipated. ATP-induced [Ca^2+^]_m_ uptake reduced significantly in si-VDAC1-treated cells. ΔF decreased ~20% in response to 1 mM ATP indicating reduced but significant rise of mitochondrial Ca^2+^ (Fig 3Bi,iii). VDAC1 is known to participate in Ca^2+^ flux across the outer membrane of mitochondria, therefore knocking down of VDAC1 attenuated Ca^2+^ entry. Interestingly, unlike control (si-Scr-treated), the potentiating effect of aspirin on mitochondrial Ca^2+^ entry was not observed in si-VDAC1-treated cells. ATP- induced [Ca^2+^]_m_ rise (decrease of fluorescence) were same with or without aspirin treatment (Fig. 3Bi,iii). Same trend was observed when ionomycin was used to elevate [Ca^2+^]_m_. Control cells showed ~70% decrease of ΔF upon ionomycin treatment, which significantly changed to ~ 90% in aspirin treated cells (Fig. 3Bii,iii). In siVDAC1-treated cells the ΔF decreased ~30% both with ionomycin and ionomycin + aspirin (Fig. 3Bii,iii). It implies that that aspirin potentiates Ca^2+^ entry to the mitochondria by acting on VDAC1.

Further, to check the involvement of Ca^2+^ in aspirin-induced cell death, cells were incubated with BAPTA-AM, a known chelator of Ca^2+^. Cell death was reduced considerably in BAPTA-treated cells (Supplementary Fig. S2).

### Aspirin dissociates mitochondrially bound HK-II but not HK-I

**Figure 4:**
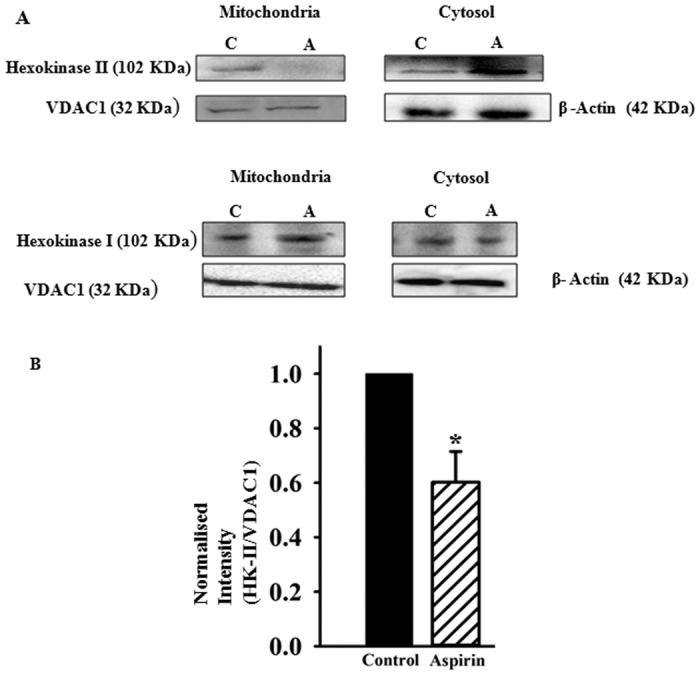
Aspirin dissociates HK-II, but not HK-I from mitochondria. (**A**) Western blot, showing the release of HK-II from mitochondria upon aspirin treatment. Cells were treated with 100 μM of aspirin for 6 h. Mitochondria and cytosolic fractions were isolated as described in ‘methods’ section. C: control; A: aspirin treated. Mitochondria, isolated from control (untreated) cells show the presence of both HK-I and HK-II. Aspirin treatment reduced the content of HK-II in mitochondria and subsequently it was increased in cytosol. In identical experimental condition, HK-I concentration did not alter either in mitochondria or in cytosol, following aspirin treatment. (**B**) Bar diagram depicts the relative quantity of HK-II in mitochondria. Band intensity was measured densitometrically and normalized in respect to the VDAC1 band intensity. Values are the mean ± SEM of three independent experiments.

Since several pro-apoptotic agents are known to release mitochondrially bound HK by disrupting VDAC-HK interaction^38^,^39^,^40^, we anticipated similar activity of aspirin. Mitochondria and cytosolic fractions were isolated from the control and aspirin treated HeLa cells. Total proteins from both mitochondrial and cytosolic fractions were resolved on SDS-PAGE and probed in Western blot using monoclonal antibody against HK-I and HK-II. VDAC1 and β actin were probed as loading control for mitochondria and cytosol respectively. Figure 4 shows that the amount of HK-II in mitochondrial fraction reduced significantly after aspirin treatment. Consequently, the cytosolic content of HK-II increased, following aspirin treatment. However, aspirin did not alter the content of HK-I either in mitochondria or in cytosol. It clearly demonstrates that aspirin releases mitochondria- associated HK-II.

### Aspirin induces closure of VDAC1

**Figure 5:**
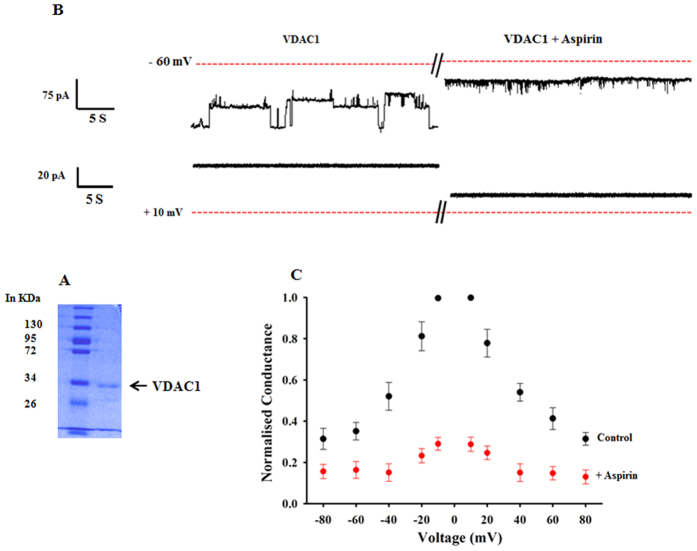
Effect of Aspirin on hVDAC1. (**A**) SDG-PAGE profile, showing the homogeneity of purified hVDAC1. (**B**) Representative current traces of VDAC1 recorded at −60 mV and +10 mV. Current value decreased when 100 μM of aspirin was added to the *cis* side of PLB. Left panel shows control hVDAC1 without aspirin treatment. Dotted lines in red represent base line (0 pA) and the holding potentials are indicated in the left. (**C**) Relative conductance (G/G_0_; G: conductance at a given voltage, G_0_: maximum conductance) versus voltage plot of VDAC1. After aspirin treatment (100 μM), channel conductance reduced. The effect was evident in all voltages. The values are the mean ± SEM of 7–10 independent experiments.

To test the direct effect of aspirin on VDAC1, recombinant human VDAC1 was overexpressed and purified. Figure 5A shows the coomassie-stained purified VDAC1 on SDS-PAGE. Purified VDAC was reconstituted in PLB and the channel properties were studied before and after aspirin treatment. Aspirin (100 μM) induced the closing of VDAC1 when added in the *cis* side of the PLB. The current traces recorded at −60 mV and + 10 mV and are shown (Fig. 5B). At 10 mV, VDAC remained fully open, but the current amplitude decreased after addition of aspirin. The single channel conductance (in 1 M KCl) at 10 mV decreased from 4.02 ± 0.24 nS, to 1.2 ± 0.18 nS upon treatment with aspirin. At −60 mV holding potential, VDAC fluctuates between open state and different closed/sub-conductance states. However after aspirin treatment VDAC stabilized in the closed state (5B). In Fig. 5C, the normalized channel conductance is plotted against voltages. As shown in the figure, aspirin reduced the channel conductance at all voltages.

## Discussion

Aspirin induces death in several types of cancer cells through apoptosis. Different mechanisms e.g. inhibition of proteasome function, cell cycle arrest and activation of caspases-8 have been shown as underlying mechanisms^12^,^13^,^14^,^15^,^16^,^17^,^18^. Here for the first time we showed that aspirin directly modulates VDAC1, leading to cell death. Aspirin-induced cell death is lesser in si-VDAC1-treated cells compared to si-Scr-treated control cells. VDAC1 plays a crucial role in apoptosis. In the intrinsic pathway of apoptosis, mitochondrial matrix remodeling is followed by the change in mitochondrial shape, reduction of Δψ_m_ and the release of cytochrome c^50^. Loss of Δψ_m_ is considered as an early event of the induction of apoptosis in many cell types^51^,^52^,^53^. We observed a time dependent loss of Δψ_m_ upon aspirin treatment. Interestingly, dissipation of Δψ_m_ is attenuated in si-VDAC1-treated cells.

The elevated [Ca^2+^]_i_ is removed from the cytosol by several means, including its uptake by mitochondria and ER. The rise of Ca^2+^ in mitochondria over a considerate period leads to apoptosis. We showed that aspirin (100 μM) augmented both ionomycin and ATP-induced [Ca^2+^]_m_ rise, and VDAC1 is involved in this process. VDAC1 is the major Ca^2+^ entry channel across the outer membrane of mitochondria. Therefore, knocking down of VDAC1 attenuated ATP-induced Ca^2+^ entry to mitochondria. Interestingly, the potentiating effect of aspirin on mitochondrial Ca^2+^ influx was also abrogated in si-VDAC1-treated cells. It implies that aspirin exerts its effect possibly by modulating VDAC1. To ascertain this, we studied the effect of aspirin on the electrophysiological properties of purified VDAC1, reconstituted in PLB. Aspirin induced the closure of VDAC1. The channel conductance reduced markedly after aspirin treatment. Closure of VDAC1 limits the normal flux of metabolites and ions, resulting in the induction of cell death processes. Additionally, in the closed state as VDAC1 is cation selective, mitochondrial Ca^2+^ influx increased which in turn triggers the events associated with apoptosis 54,55. Several other pro-apoptotic agents have also been reported to induce VDAC1- closure^56^,^57^.

Many cancer cells adapt a survival mechanism in the hostile hypoxic micro-environment by translocating HK-II to the mitochondria. Interaction of HK-II and VDAC1 provides metabolic advantage to the cancer cells by strengthening anaerobic glycolysis. When HK-II was dissociated from mitochondria, cells became sensitive to many apoptotic agents^58^. Many anti-cancer compounds have been shown to release HK-II from mitochondria. We showed that aspirin dissociates HK-II from mitochondria in intact HeLa cells. However, it is not clear if the desorption of HK-II is solely due the direct interaction of aspirin with VDAC1. Interaction of aspirin with HK-II cannot be ruled out. Therefore, aspirin-induced cell death is a cumulative effect of VDAC1-closure and desorption of HK-II from mitochondria. Disruption of mitochondrial calcium homeostasis and dissipation of Δψ_m,_ which aid to the cell death process are possibly the outcome of VDAC-closure. In summary, we have reported VDAC1 as a new target for aspirin. Aspirin-induced closure of VDAC1 correlates with the elevation of mitochondrial Ca^2+^, a strong apoptotic signal. Additionally, aspirin dissociated HK-II from mitochondria that cumulatively decreased cell viability. Our observations will be helpful in designing aspirin based anti-cancer drugs.

## Materials and Methods

### Materials

HeLa and SCC131 cells were obtained from National Centre for Cell Sciences, Pune, India. FBM cell line was kindly gifted by Dr. Milind Vaidya (ACTREC, India). Dulbecco’s modified Eagle’s medium (DMEM), fetal bovine serum (FBS), penicillin, streptomycin were purchased from Hi Media, India. *E. coli* M15 bacterial strain and Ni-NTA matrix were purchased from Qiagen. All the salts were purchased from Sigma-Aldrich. Fura 2-AM, and JC-1 were purchased from Molecular probes Inc., USA. Antibodies were procured from Cell Signaling Technologies (CST), USA. Inverse pericam construct was kindly gifted by Dr. Atsushi, Miyawaki Riken, Japan.

### Purification and reconstitution of human VDAC1

The plasmid PDS56/RBII-6xHis encoding His tag human VDAC1 (hVDAC1) was transformed in *E.Coli* M15 (pREP4). The over-expressed protein was purified using Ni-NTA column as described before^43^. Purified VDAC was reconstituted in the PLB, made up of 1,2- diphytanoyl-sn-glycero-3 phosphatidyl choline (DPhPC) (Avanti Polar Lipids, Alabaster, AL), following the method described earlier^59^. Briefly, DPhPC (20 mg/ml in n-decane) was painted on the 150 μm diameter aperture of a polystyrene bilayer cuvette (Warner instrument, USA). Both *cis* and *trans* chambers were filled with symmetrical solutions of 1 M KCl, 5 mM MgCl_2_ and 10 mM HEPES (pH 7.4). *Cis* chamber was connected to the ground electrode and *trans* chamber was connected to the amplifier through PC501A headstage (Warner Instrument, USA). Bilayer formation was monitored by measuring the membrane capacitance. Purified VDAC was added to the *cis* chamber and the solution was mixed with magnetic stirrer. Channel activity was recorded at different voltages before and after adding aspirin (100 μM final concentration) to the *cis* chamber. Currents were low pass filtered at 1 kHz and digitized at 5 kHz. The pClamp software (version 9, Molecular Devices) was used for data acquisition and analysis.

### siRNA knockdown of VDAC1

Scrambled and human specific hVDAC1 siRNAs were obtained from Sigma Aldrich. HeLa cells were seeded on six-well culture dishes. 50-70% confluent cells were transfected with different amount of hVDAC1 siRNA, using Lipofectamine reagent (Life Technologies), according to the protocol provided by the manufacturer.

### SDS-PAGE and Western blotting

Cells were lysed in PBS, supplemented with protease inhibitor cocktail. Approximately 50 μg of total protein was resolved on 12% SDS-PAGE and then transferred to polyvinylidene fluoride (PVDF) membrane (Bio-Rad, USA)^60^. The blocking was done with 5% BSA for 1 h at room temperature and then the blots were incubated overnight at 4 °C with different primary antibodies. Antibodies against hVDAC1 (CST catalogue #4866 S) and HK-II, HK-I (CST catalogue # C64G5, C35C4 respectively) were diluted to 1:750; for cytochrome c (CST catalogue # 136F3), 1:1000 dilution and for β actin, 1:2000 dilution were used. After several washes with TBS-Tween-20 solution, the blots were incubated for 1 h with HRP-conjugated secondary antibody at 1:5000 dilution. The blots were treated with super signal west pico-chemiluminescent substrate (Thermo Scientific, USA) and then visualized on a Chemidoc XRS (Bio-Rad).

### Measurement of mitochondrial membrane potential (Δψ_m_)

Cells were incubated with 0.5 μM of JC-1 dye for 15 min at 37 °C and then washed with PBS. Glass coverslip containing cells was placed in an imaging chamber and perfused continuously with the bathing solution (pH 7.4) containing (in mM): NaCl 126, KCl 4, NaHCO_3_ 26, CaCl_2_ 1.8, NaH_2_PO_4_ 1.5, MgSO_4_ 1.5 and Glucose 10. Using appropriate filter set up, cells were excited at 488 nm and the emission was captured at 534 nm & 596 nm. Images were taken at 5 min interval with Andor EMCCD camera, attached with an inverted microscope (Olympus IX71). The intensities of the red (R) and green (G) fluorescence were calculated from the background subtracted images. Fluorescence ratio (R/G) was plotted against time.

### Measurement of cytosolic and mitochondrial Ca^2+^

Cytosolic Ca^2+^ was estimated ratiometrically using fura-2AM as described before^61^. Briefly, cells were incubated with 10 μM fura 2-AM (Invitrogen, USA) in bath solution at room temperature for 30 min. Cells were washed for 30 min in fura-free solution. Coverslip containing fura-loaded cells were placed in a small glass bottom recording chamber, mounted on the stage of the Olympus inverted microscope (IX71). Cells were illuminated alternatively with 340 nm & 380 nm, with the help of Lambda-DG4 (Sutter instruments, USA), and the emission was set to 510 nm. Images were acquired at every 5 second interval. F_340_/F_380_ was calculated from the background subtracted images using Andor IQ software.

Mitochondrial Ca^2+^ was measured in the cells transfected with GFP based Ca^2+^ sensor, inverse pericam^62^. Fluorescence intensity (ΔF) of the inverse pericam decreases with increasing concentration of mitochondrial Ca^2+^. Images were acquired every 5 seconds and the intensity was calculated off line using Andor IQ program. Ca^2+^ rise was triggered with 1 mM ATP or 10 μM ionomycin. To see the effect of aspirin, cells were pre-treated with 100 μM aspirin for 10 min.

### Isolation of mitochondria from HeLa cells

Mitochondria were isolated from HeLa cells as described earlier^63^. Briefly, HeLa cells, grown in flask were rinsed with PBS. Then cells were lifted by scrapping. Cells were centrifuged at 2000× g for 5 min and the pellet obtained was homogenized with 200 μl IBC buffer (2: 225-mM mannitol, 75-mM sucrose and 30-mM Tris–HCl pH 7.4) in ice using a Dounce homogenizer. The sample was centrifuged at 4500× g for 10 min at 4 °C. The supernatant was collected and again centrifuged at 10000× g for 20 min at 4 °C. The mitochondrial pellet was stored at −80 °C for future use (for immunoblot experiments).

### MTT assay

Cell viability was estimated by MTT assay. The cells were treated with 3-(4, 5-Dimethylthiazol-2-yl)−2, 5-diphenyltetrazolium bromide (MTT) for 3 h at room temperature in dark. The dye was solubilized with acidified isopropanol, followed by centrifugation. The absorbance of the supernatant was monitored at 570 nm.

### Statistical analysis

Student’s t-test was used to compare two groups. One way ANOVA was performed for comparing several groups. P values less than 0.05 were considered as significant difference.

## Additional Information

**How to cite this article:** Tewari, D. *et al*. Aspirin induces cell death by directly modulating mitochondrial voltage-dependent anion channel (VDAC). *Sci. Rep.* **7**, 45184; doi: 10.1038/srep45184 (2017).

**Publisher's note:** Springer Nature remains neutral with regard to jurisdictional claims in published maps and institutional affiliations.

## Supplementary Material

Supplementary Information

## References

[b1] Brune K. & Patrignani P. New insights into the use of currently available non-steroidal anti-inflammatory drugs. J Pain Res 8, 105–118, doi: 10.2147/JPR.S75160jpr-8-105 (2015). 25759598 PMC4346004

[b2] Dinarello C. A. Anti-inflammatory Agents: Present and Future. Cell 140, 935–950, doi: 10.1016/j.cell.2010.02.043S0092-8674(10)00236-9 (2010). 20303881 PMC3752337

[b3] Vane J. R. & Botting R. M. The mechanism of action of aspirin. Thromb Res 110, 255–258, doi: S0049384803003797 (2003). 14592543 10.1016/s0049-3848(03)00379-7

[b4] Cohen A. T., Imfeld S., Markham J. & Granziera S. The use of aspirin for primary and secondary prevention in venous thromboembolism and other cardiovascular disorders. Thromb Res 135, 217–225, doi: 10.1016/j.thromres.2014.11.036S0049-3848(14)00672-0 (2015). 25541030

[b5] Hennekens C. H. & Dalen J. E. Aspirin in the treatment and prevention of cardiovascular disease: past and current perspectives and future directions. Am J Med 126, 373–378, doi: 10.1016/j.amjmed.2012.12.013S0002-9343(13)00096-X (2013). 23499330

[b6] Pinto A., Di Raimondo D., Tuttolomondo A., Butta C. & Licata G. Antiplatelets in stroke prevention. Curr Vasc Pharmacol 11, 803–811, doi: CVP-58818 (2013). 24484461 10.2174/157016111106140128112915

[b7] Alfonso L., Ai G., Spitale R. C. & Bhat G. J. Molecular targets of aspirin and cancer prevention. Br J Cancer 111, 61–67, doi: 10.1038/bjc.2014.271bjc2014271 (2014). 24874482 PMC4090734

[b8] Garcia-Albeniz X. & Chan A. T. Aspirin for the prevention of colorectal cancer. Best Pract Res Clin Gastroenterol 25, 461–472, doi: 10.1016/j.bpg.2011.10.015S1521-6918(11)00103-X (2011). 22122763 PMC3354696

[b9] Maity G. . Aspirin blocks growth of breast tumor cells and tumor-initiating cells and induces reprogramming factors of mesenchymal to epithelial transition. Lab Invest 95, 702–717, doi: 10.1038/labinvest.2015.49labinvest201549 (2015). 25867761

[b10] Muranushi C., Olsen C. M., Pandeya N. & Green A. C. Aspirin and nonsteroidal anti-inflammatory drugs can prevent cutaneous squamous cell carcinoma: a systematic review and meta-analysis. J Invest Dermatol 135, 975–983, doi: 10.1038/jid.2014.531S0022-202X(15)37185-2 (2015). 25521453

[b11] Usman M. W., Luo F., Cheng H., Zhao J. J. & Liu P. Chemopreventive effects of aspirin at a glance. Biochim Biophys Acta 1855, 254–263, doi: 10.1016/j.bbcan.2015.03.007S0304-419X(15)00022-0 (2015). 25842298

[b12] Bellosillo B. . Aspirin and salicylate induce apoptosis and activation of caspases in B-cell chronic lymphocytic leukemia cells. Blood 92, 1406–1414 (1998). 9694730

[b13] Dikshit P., Chatterjee M., Goswami A., Mishra A. & Jana N. R. Aspirin induces apoptosis through the inhibition of proteasome function. J Biol Chem 281, 29228–29235, doi: M602629200 10.1074/jbc.M602629200 (2006). 16880202

[b14] Klampfer L., Cammenga J., Wisniewski H. G. & Nimer S. D. Sodium salicylate activates caspases and induces apoptosis of myeloid leukemia cell lines. Blood 93, 2386–2394 (1999). 10090950

[b15] Stark L. A., Din F. V., Zwacka R. M. & Dunlop M. G. Aspirin-induced activation of the NF-kappaB signaling pathway: a novel mechanism for aspirin-mediated apoptosis in colon cancer cells. FASEB J 15, 1273–1275 (2001). 11344111

[b16] Gu Q. . Activation of the caspase-8/Bid and Bax pathways in aspirin-induced apoptosis in gastric cancer. Carcinogenesis 26, 541–546, doi: bgh345 10.1093/carcin/bgh345 (2005). 15579484

[b17] Pathi S. . Aspirin inhibits colon cancer cell and tumor growth and downregulates specificity protein (Sp) transcription factors. PLoS One 7, e48208, doi: 10.1371/journal.pone.0048208PONE-D-12-23952 (2012). 23110215 PMC3482208

[b18] Stark L. A. . Aspirin activates the NF-kappaB signalling pathway and induces apoptosis in intestinal neoplasia in two *in vivo* models of human colorectal cancer. Carcinogenesis 28, 968–976, doi: bgl220 10.1093/carcin/bgl220 (2007). 17132819

[b19] Gincel D., Zaid H. & Shoshan-Barmatz V. Calcium binding and translocation by the voltage-dependent anion channel: a possible regulatory mechanism in mitochondrial function. Biochem J 358, 147–155 (2001). 11485562 10.1042/0264-6021:3580147 PMC1222042

[b20] Shoshan-Barmatz V. & Ben-Hail D. VDAC, a multi-functional mitochondrial protein as a pharmacological target. Mitochondrion 12, 24–34, doi: 10.1016/j.mito.2011.04.001S1567-7249(11)00182-6 (2012). 21530686

[b21] Colombini M. Voltage gating in the mitochondrial channel, VDAC. J Membr Biol 111, 103–111 (1989). 2482359 10.1007/BF01871775

[b22] Colombini M. VDAC structure, selectivity, and dynamics. Biochim Biophys Acta 1818, 1457–1465, doi: 10.1016/j.bbamem.2011.12.026S0005-2736(11)00463-9 (2012). PMC3327780 22240010

[b23] Rostovtseva T. K., Tan W. & Colombini M. On the role of VDAC in apoptosis: fact and fiction. J Bioenerg Biomembr 37, 129–142, doi: 10.1007/s10863-005-6566-8 (2005). 16167170

[b24] Baumgartner H. K. . Calcium elevation in mitochondria is the main Ca^2+^ requirement for mitochondrial permeability transition pore (mPTP) opening. J Biol Chem 284, 20796–20803, doi: 10.1074/jbc.M109.025353M109.025353 (2009). 19515844 PMC2742844

[b25] Feldmann G. . Opening of the mitochondrial permeability transition pore causes matrix expansion and outer membrane rupture in Fas-mediated hepatic apoptosis in mice. Hepatology 31, 674–683, doi: S0270913900637527 10.1002/hep.510310318 (2000). 10706558

[b26] Wang L., Yang X. & Shen Y. Molecular mechanism of mitochondrial calcium uptake. Cell Mol Life Sci 72, 1489–1498, doi: 10.1007/s00018-014-1810-1 (2015). 25548802 PMC11113575

[b27] Baines C. P., Kaiser R. A., Sheiko T., Craigen W. J. & Molkentin J. D. Voltage-dependent anion channels are dispensable for mitochondrial-dependent cell death. Nat Cell Biol 9, 550–555, doi: ncb1575 10.1038/ncb1575 (2007). 17417626 PMC2680246

[b28] Krauskopf A., Eriksson O., Craigen W. J., Forte M. A. & Bernardi P. Properties of the permeability transition in VDAC1(−/−) mitochondria. Biochim Biophys Acta 1757, 590–595, doi: S0005-2728(06)00033-8 10.1016/j.bbabio.2006.02.007 (2006). 16626625

[b29] De Stefani D. . VDAC1 selectively transfers apoptotic Ca^2+^ signals to mitochondria. Cell Death Differ 19, 267–273, doi: 10.1038/cdd.2011.92cdd201192 (2012). 21720385 PMC3263501

[b30] Arbel N., Ben-Hail D. & Shoshan-Barmatz V. Mediation of the antiapoptotic activity of Bcl-xL protein upon interaction with VDAC1 protein. J Biol Chem 287, 23152–23161, doi: 10.1074/jbc.M112.345918 M112.345918 (2012). 22589539 PMC3391160

[b31] Carre M. . Tubulin is an inherent component of mitochondrial membranes that interacts with the voltage-dependent anion channel. J Biol Chem 277, 33664–33669, doi: 10.1074/jbc.M203834200M203834200 (2002). 12087096

[b32] Pastorino J. G. & Hoek J. B. Regulation of hexokinase binding to VDAC. J Bioenerg Biomembr 40, 171–182, doi: 10.1007/s10863-008-9148-8 (2008). 18683036 PMC2662512

[b33] Tsujimoto Y. & Shimizu S. VDAC regulation by the Bcl-2 family of proteins. Cell Death Differ 7, 1174–1181, doi: 10.1038/sj.cdd.4400780 (2000). 11175254

[b34] Xu X., Forbes J. G. & Colombini M. Actin modulates the gating of Neurospora crassa VDAC. J Membr Biol 180, 73–81 (2001). 11284205 10.1007/s002320010060

[b35] Mathupala S. P., Ko Y. H. & Pedersen P. L. Hexokinase II: cancer’s double-edged sword acting as both facilitator and gatekeeper of malignancy when bound to mitochondria. Oncogene 25, 4777–4786, doi: 1209603 10.1038/sj.onc.1209603 (2006). 16892090 PMC3385868

[b36] Pedersen P. L., Mathupala S., Rempel A., Geschwind J. F. & Ko Y. H. Mitochondrial bound type II hexokinase: a key player in the growth and survival of many cancers and an ideal prospect for therapeutic intervention. Biochim Biophys Acta 1555, 14–20, doi: S0005272802002487 (2002). 12206885 10.1016/s0005-2728(02)00248-7

[b37] Pedersen P. L. Voltage dependent anion channels (VDACs): a brief introduction with a focus on the outer mitochondrial compartment’s roles together with hexokinase-2 in the “Warburg effect” in cancer. J Bioenerg Biomembr 40, 123–126, doi: 10.1007/s10863-008-9165-7 (2008). 18780167

[b38] Galluzzi L., Kepp O., Tajeddine N. & Kroemer G. Disruption of the hexokinase-VDAC complex for tumor therapy. Oncogene 27, 4633–4635, doi: 10.1038/onc.2008.114onc2008114 (2008). 18469866

[b39] Goldin N. . Methyl jasmonate binds to and detaches mitochondria-bound hexokinase. Oncogene 27, 4636–4643, doi: 10.1038/onc.2008.108onc2008108 (2008). 18408762

[b40] Arzoine L., Zilberberg N., Ben-Romano R. & Shoshan-Barmatz V. Voltage-dependent anion channel 1-based peptides interact with hexokinase to prevent its anti-apoptotic activity. J Biol Chem 284, 3946–3955, doi: 10.1074/jbc.M803614200M803614200 (2009). 19049977

[b41] Prezma T. . VDAC1-based peptides: novel pro-apoptotic agents and potential therapeutics for B-cell chronic lymphocytic leukemia. Cell Death Dis 4, e809, doi: 10.1038/cddis.2013.316cddis2013316 (2013). 24052077 PMC3789174

[b42] Rimmerman N. . Direct modulation of the outer mitochondrial membrane channel, voltage-dependent anion channel 1 (VDAC1) by cannabidiol: a novel mechanism for cannabinoid-induced cell death. Cell Death Dis 4, e949, doi: 10.1038/cddis.2013.471cddis2013471 (2013). 24309936 PMC3877544

[b43] Tewari D. . Modulation of the mitochondrial voltage-dependent anion channel (VDAC) by curcumin. Biochim Biophys Acta 1848, 151–158, doi: 10.1016/j.bbamem.2014.10.014S0005-2736(14)00349-6 (2015). 25459681

[b44] Zheng Y. . Essential role of the voltage-dependent anion channel (VDAC) in mitochondrial permeability transition pore opening and cytochrome c release induced by arsenic trioxide. Oncogene 23, 1239–1247, doi: 10.1038/sj.onc.12072051207205 (2004). 14647451 PMC2913247

[b45] Pique M. . Aspirin induces apoptosis through mitochondrial cytochrome c release. FEBS Lett 480, 193–196, doi: S0014-5793(00)01922-0(2000). 10.1016/s0014-5793(00)01922-0 11034327

[b46] Zimmermann K. C., Waterhouse N. J., Goldstein J. C., Schuler M. & Green D. R. Aspirin induces apoptosis through release of cytochrome c from mitochondria. Neoplasia 2, 505–513 (2000). 11228543 10.1038/sj.neo.7900120 PMC1508093

[b47] Flescher E. . Aspirin-like drugs prime human T cells. Modulation of intracellular calcium concentrations. J Immunol 146, 2553–2559 (1991). 1901879

[b48] Levine R. A., Nandi J. & King R. L. Aspirin potentiates prestimulated acid secretion and mobilizes intracellular calcium in rabbit parietal cells. J Clin Invest 86, 400–408, doi: 10.1172/JCI114725 (1990). 2166752 PMC296741

[b49] Welter-Stahl L. . Expression of purinergic receptors and modulation of P2X7 function by the inflammatory cytokine IFNgamma in human epithelial cells. Biochim Biophys Acta 1788, 1176–1187, doi: 10.1016/j.bbamem.2009.03.006S0005-2736(09)00082-0 (2009). 19306841

[b50] Gottlieb E., Armour S. M., Harris M. H. & Thompson C. B. Mitochondrial membrane potential regulates matrix configuration and cytochrome c release during apoptosis. Cell Death Differ 10, 709–717, doi: 10.1038/sj.cdd.4401231 4401231 (2003). 12761579

[b51] Bortner C. D. & Cidlowski J. A. Caspase independent/dependent regulation of K(+), cell shrinkage, and mitochondrial membrane potential during lymphocyte apoptosis. J Biol Chem 274, 21953–21962 (1999). 10419518 10.1074/jbc.274.31.21953

[b52] Chun Y.-J., Seung-Hoonlee S.-I. & Rhee C. H. Arsenic trioxide induces apoptosis through a reactive oxygen species-dependent pathway and loss of mitochondrial membrane potential in HeLa cells. Int.J.Oncol. 21, 57–63 (2002). 12063550

[b53] Cook S. A., Sugden P. H. & Clerk A. Regulation of bcl-2 family proteins during development and in response to oxidative stress in cardiac myocytes: association with changes in mitochondrial membrane potential. Circ Res 85, 940–949 (1999). 10559141 10.1161/01.res.85.10.940

[b54] Hodge T. & Colombini M. Regulation of metabolite flux through voltage-gating of VDAC channels. J Membr Biol 157, 271–279 (1997). 9178614 10.1007/s002329900235

[b55] Tan W. & Colombini M. VDAC closure increases calcium ion flux. Biochim Biophys Acta 1768, 2510–2515, doi: S0005-2736(07)00213-1 10.1016/j.bbamem.2007.06.002 (2007). 17617374 PMC2220155

[b56] McCommis K. S. & Baines C. P. The role of VDAC in cell death: friend or foe? Biochim Biophys Acta 1818, 1444–1450, doi: 10.1016/j.bbamem.2011.10.025S0005-2736(11)00375-0 (2012). 22062421 PMC3288473

[b57] Tan W. VDAC blockage by phosphorothioate oligonucleotides and its implication in apoptosis. Biochim Biophys Acta 1818, 1555–1561, doi: 10.1016/j.bbamem.2011.12.032S0005-2736(12)00002-8 (2012). 22236836

[b58] Chiara F. . Hexokinase II detachment from mitochondria triggers apoptosis through the permeability transition pore independent of voltage-dependent anion channels. PLoS One 3, e1852, doi: 10.1371/journal.pone.0001852 (2008). 18350175 PMC2267038

[b59] Bera A. K., Ghosh S. & Das S. Mitochondrial VDAC can be phosphorylated by cyclic AMP-dependent protein kinase. Biochem Biophys Res Commun 209, 213–217, doi: S0006-291X(85)71491-X 10.1006/bbrc.1995.1491 (1995). 7537039

[b60] Laemmli U. K. Cleavage of structural proteins during the assembly of the head of bacteriophage T4. Nature 227, 680–685 (1970). 5432063 10.1038/227680a0

[b61] Sahu G. & Bera A. K. Contribution of intracellular calcium and pH in ischemic uncoupling of cardiac gap junction channels formed of connexins 43, 40, and 45: a critical function of C-terminal domain. PLoS One 8, e60506, doi: 10.1371/journal.pone.0060506PONE-D-12-39940 (2013). 23536911 PMC3607587

[b62] Robert V. . Beat-to-beat oscillations of mitochondrial [Ca^2+^] in cardiac cells. EMBO J 20, 4998–5007, doi: 10.1093/emboj/20.17.4998 (2001). 11532963 PMC125611

[b63] Wieckowski M. R., Giorgi C., Lebiedzinska M., Duszynski J. & Pinton P. Isolation of mitochondria-associated membranes and mitochondria from animal tissues and cells. Nat Protoc 4, 1582–1590, doi: 10.1038/nprot.2009.151nprot.2009.151 (2009). 19816421

