# Supplementary material for: Aspirin induces cell death by directly modulating mitochondrial voltage-dependent anion channel (VDAC)
Source: Sci Rep. 2017 Mar 22;7:45184. doi: 10.1038/srep45184 (PMC5361111; doi:10.1038/srep45184)
Supplement: Supplementary Information [file srep45184-s1.doc]

**Supplementary Information**

**Aspirin induces cell death by directly modulating mitochondrial voltage-dependent anion channel (VDAC)**

Debanjan Tewari #, Dhriti Majumdar #, Sirisha Vallabhaneni, Amal Kanti Bera *

**Aspirin induces cell death by directly modulating mitochondrial voltage-dependent anion channel (VDAC)**

**Fig. S1**


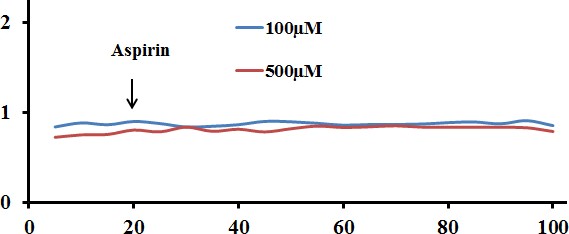


**Aspirin induces cell death by directly modulating mitochondrial voltage-dependent anion channel (VDAC)**


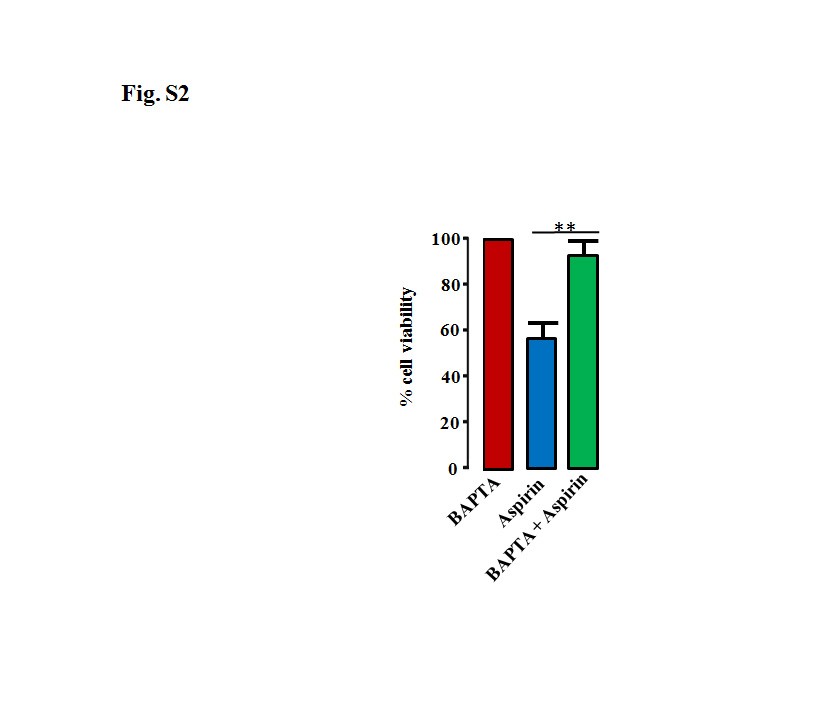


**Aspirin induces cell death by directly modulating mitochondrial voltage-dependent anion channel (VDAC)**

**Supplementary figure legends**

**Fig. S1: 100 μM and 500 μM aspirin alone do not change cytosolic [Ca^2+^]_i_ in HeLa cell.**

**Fig.S2: Calcium chelator BAPTA prevents aspirin-induced cell death.**

HeLa cells were incubated with or without 3 μM BAPTA-AM prior to the aspirin (100 μM)

treatment. Cells pre-treated with BAPTA-AM showed significantly lesser cell death by aspirin, compared to the cells treated with aspirin alone. ** p< 0.01.
